# Differential Host Immune Responses to Epidemic and Endemic Strains of *Shigella dysenteriae* Type 1

**DOI:** 10.3329/jhpn.v29i5.8896

**Published:** 2011-10

**Authors:** Mohammad Abu Sayem, Shaikh Meshbahuddin Ahmad, Rokeya Sultana Rekha, Protim Sarker, Birgitta Agerberth, Kaisar Ali Talukder, Rubhana Raqib

**Affiliations:** ^1^Laboratory Sciences Division, icddr,b, GPO Box 128, Dhaka 1000, Bangladesh; ^2^Department of Biotechnology and Genetic Engineering, Mawlana Bhashani Science and Technology University, Tangail, Bangladesh; ^3^Department of Medical Biochemistry and Biophysics, Karolinska Institutet, Stockholm, Sweden

**Keywords:** Disease models, Animal, Dysentery, Bacillary, Immune response, *Shigella dysenteriae*

## Abstract

*Shigella dysenteriae* type 1 causes devastating epidemics in developing countries with high case-fatality rates in all age-groups. The aim of the study was to compare host immune responses to epidemic (T2218) and endemic strains of *S. dysenteriae* type 1. Shigellacidal activity of serum from rabbits immunized with epidemic or endemic strains, *S. dysenteriae* type 1-infected patients, and healthy adult controls from *Shigella* endemic and non-endemic regions was measured. Immunogenic cross-reactivity of antibodies against *Shigella* antigens was evaluated by Western blot analysis. Oxidative burst and phagocytic responses of monocytes and neutrophils to selected *S. dysenteriae* type 1 strains were assessed by flow cytometry. Rabbit antisera against epidemic strain were less effective in killing heterologous bacteria compared to endemic antisera (p=0.0002). Patients showed an increased serum shigellacidal response after two weeks of onset of diarrhoea compared to the acute stage (3-4 days after onset) against their respective homologous strains; the response against T2218 and heterologous endemic *S. dysenteriae* type 1 strains was not significant. The serum shigellacidal response against all the *S. dysenteriae* type 1 strains was similar among healthy controls from endemic and non-endemic regions and was comparable with the acute stage response by patients. Compared to endemic strains of *S. dysenteriae* type 1, T2218 was significantly resistant to phagocytosis by both monocytes and neutrophils. No obvious differences were obtained in the induction of oxidative burst activity and cathelicidin-mediated killing. Cross-reactivity of antibody against antigens present in the epidemic and endemic strains showed some differences in protein/peptide complexity and intensity by Western blot analysis. In summary, epidemic T2218 strain was more resistant to antibody-mediated defenses, namely phagocytosis and shigellacidal activity, compared to endemic *S. dysenteriae* type 1 strains. Part of this variation may be attributed to the differential complexity of protein/peptide antigens.

## INTRODUCTION

Shigellosis is a global public-health problem. According to the recent report of the World Health Organization on the worldwide burden of shigellosis, 90 million cases of diarrhoea due to *Shigella* occur annually mostly in developing countries, and an estimated 108,000 deaths per year are attributed to *Shigella* infections (http://www.who.int/vaccine_research/diseases/diarrhoeal/en/index6.html # vaccine). Currently, no licensed vaccines against *Shigella* infection exist (1).

Four species of *Shigella* are responsible for causing bacillary dysentery: *Shigella dysenteriae, S. flexneri, S. sonnei*, and *S. boydii*. Of the four species, *S. dysenteriae* type 1 produces the most severe form of disease with a high mortality rate in young children, elderly people, and the malnourished and can be associated with various complications (1,2). Since the 1960s, *S. dysenteriae* type 1 is known to be an important cause of epidemic dysentery in Latin America, Africa, and Asia, including Bangladesh (1,3-6). Epidemic shigellosis largely depends on drug resistance (7), variation in Shiga toxin (8), or other unknown causes. Studies on differences between epidemic and circulating endemic strains of *Shigella* are scarce. In one study, the plasmid profile of *Shigella* spp. isolated during an outbreak was shown to be different from that of endemic isolates (9). Another study reported that, during sporadic and outbreak incidences of shigellosis, epidemic variants had the most stable plasmids (10). To understand the transmission dynamics of *Vibrio cholerae* during epidemics, Merrel *et al*. showed that increased infectivity of *V. cholerae* was associated with variable expression level of various genes (11). However, till date, no studies on comparison between host immune responses to epidemic and endemic *Shigella* isolates have been conducted.

Studies to understand the differences in the host immune response to epidemic and endemic *Shigella* strains may eventually aid in designing alternative treatment strategies during epidemics and vaccine development. Therefore, we aimed to assess the immune responses evoked by epidemic and endemic strains of *S. dysenteriae* type 1 by studying phagocytic response and oxidative burst of neutrophils and monocytes, serum and LL-37-mediated killing of these strains. The difference in immunogenicity of these strains was also evaluated by Western blot analysis of the whole-cell antigens.

## MATERIALS AND METHODS

### *S. dysenteriae* type 1 strains

The epidemic strain T2218 was collected during a dysentery epidemic in Teknaf, a coastal area in Bangladesh, that caused high mortality among children aged less than one year (12). Endemic *S. dysenteriae* type 1 strains isolated from *S. dysentetiae* type 1-infected adult patients (n=8) admitted to the Dhaka Hospital of icddr,b enrolled in a previous study (13) were used in the present study. Patients were empirically treated with pivmecillinam.

Table 1 shows the different strains that were used for various immunological assays. Serum and strain from the same source (patient or immunized rabbit) were designated as homologous while serum and strain isolated from different sources (patient or immunized rabbit) were designated as heterologous strain or heterologous serum. The bacterial cultures were stored at −80 °C in Trypticase Soy Broth (TSB) (Difco, Spartus, MD) with 15% glycerol. Before using in immunological assays, isolates were confirmed by biochemical reactions, agglutination with specific antiserum, and Séreny test.

### Blood and serum samples

Patients with shigellosis were followed for one month, and blood samples were collected at different intervals after the onset of diarrhoea (3-5, 14-16, and 30-35 days) (13). For convenience, these follow-up days were referred to as days after admission (day 1, 11, and 30). Blood samples obtained from healthy adults (laboratory staff, n=15, aged 24-30 years) at icddr,b were from *Shigella*-endemic area and from healthy Swedish adults (n=5, aged 21-56 years) as *Shigella* non-endemic area (13).

### Preparation of rabbit antisera against epidemic and endemic *S. dysenteriae* type 1 strains

Permission was obtained from the Animal Ethics Committee of icddr,b for raising antisera against the bacterial strains. The *S. dysenteriae* type 1 epidemic strain (T2218) and one endemic *S. dysenteriae* type 1 strain (191316) were used for immunizing rabbits. New Zealand White rabbits (n=3 for each strain) were immunized subcutaneously and intramuscularly five times with formalin-killed *Shigella* suspension at two-day intervals using 5-10×10^7^ colony-forming units per time point. Seven to 10 days after the end of the immunization protocol, venous blood was obtained to collect serum, and antibody levels were tested by standard agglutination test (14).

### Serum shigellacidal assay

The serum shigellacidal assay was carried out as described earlier (14). In brief, bacterial strains in Mueller Hinton Broth (MHB) (Difco) (1×10^3^ cfu/mL), guinea pig complement (Sigma-Aldrich, St Louis, USA), and two-fold serially-diluted heat-inactivated serum samples (starting dilution for patient sera 1:10; for rabbit sera 1:1) were incubated in a shaker incubator (200 rpm) at 37 °C for 16 hours. The optical density was measured at 595 nm. The shigellacidal antibody titre was defined as the reciprocal of the highest serum dilution to yield >95% reduction of optical density compared to control wells without serum. The various combinations of rabbit antisera with bacteria are given in Table 2.

### Fluorescence labelling of *Shigella*

Both 191316 and T2218 strains were labelled with fluorescein isothiocyanate (FITC) (Sigma) as described previously (15,16) with minor modifications. FITC (1 mg/mL) was added to bacteria (1×10^8^/mL), incubated at room temperature for 25 minutes in a shaker, washed with phosphate buffer saline (PBS), fixed with 1% formaldehyde in dark for 30 minutes, and after washing, the labelled *Shigella* was kept at 4 ^0^C. For the *in vitro* assays, freshly-labelled bacteria were used.

### Phagocytosis and oxidative burst activity of monocytes and granulocytes by flow cytometry

Phagocytic activity was measured as previously described (17). Heparinized blood was incubated first with FITC-labelled *Shigella* (1×10^7^) (endemic *S. dysenteriae* type 1 191316 or T2218 strain) for 30 minutes at 37 ^0^C in dark and then with ethidium bromide (EtBr) (Invitrogen Corporation; Carlsbad, CA) in ice. After washing, RBC was lysed by ammonium chloride solution, and fluorescence intensity was measured by flow cytometry using the CELLQUEST software. Cells were identified by Forward scatter-Side scatter gating, and green FITC signals were detected in the FL-1 (fluorochrome 1) detector functioning at 480 nm detecting green fluorescence.

**Table 1.** List of isolates used

Isolate	Place of isolation	Year	Assays
Epidemic			
T2218	Teknaf, Bangladesh	1985	All
Endemic			
191316	Dhaka Hospital, icddr,b	2002	Phagocytosis, oxidative burst, raising antisera, and Western blot analysis
Endemic	Dhaka Hospital, icddr,b	2002	Shigellacidal assay with sera of patients
192789			
179370			
179268			
178042			
184223			
175560			
191309			
191325			
Endemic			
191316	Dhaka Hospital, icddr,b	2002	Shigellacidal assay with healthy adult and rabbit antisera
191309			
191325			

Oxidative burst activity was assessed by measuring the intensity of the redox indicator dye Di-chloro-fluorescence (DCF) (15). The intensity of DCF is an indirect measure of the level of reactive oxygen species (ROS) generated in a cell following activation. Heparinized blood was incubated first with DCFH-DA (Sigma) and then with 1×10^7^*Shigella* (endemic *S. dysenteriae* type 1 191316 or T2218 strain) for 30 minutes each at 37 ^0^C in dark. After lysing RBC with ammonium chloride, fluorescence intensity was assessed as above. DCF signals were detected in the FL-1 detector.

Controls included granulocytes in PBS without labelled bacteria and bacteria without granulocytes. In all the cases, the geometric mean fluorescence intensity (MFI) of the gated cell populations was estimated. Each assay was done in triplicates using eight different blood donors.

**Table 2.** Comparison of shigellacidal activity of rabbit antisera against epidemic and endemic *Shigella dysenteriae* type 1 strains in various combinations by Kaplan-Meier survival curve analysis

Combination	95% CI	Log-rank test	p value
EnSd1 + anti-EnSd1	0.19-0.52		
EpSd1 + anti-EpSd1	0.22-0.64	1.17	0.28
EnSd1 + anti-EnSd1	0.19-0.52		
EnSd1 + anti-EpSd1	1.0-1.0	20.1	0.00001
EpSd1 + anti-EnSd1	0.22-0.64		
EpSd1 + anti-EpSd1	0.22-0.64	0	1.0
EnSd1 + anti-EpSd1	1.0-1.0		
EpSd1 + anti-EpSd1	0.22-0.64	14.25	0.0002
EnSd1 + anti-EnSd1	0.19-0.52		
EpSd1 + anti-EnSd1	0.22-0.64	1.17	0.28

Data (1/dilution of antisera) given as 95% CI of means. Data shown here are for one endemic strain only (#191316).

CI=Confidence interval;

EnSd1=Endemic *Shigella dysenteriae* type 1 strain; EpSd1=Epidemic *S. dysenteriae* type 1 strain;

anti-EnSd1=Rabbit antisera against endemic *S. dysenteriae* type 1;

anti-EpSd1 Rabbit antisera against epidemic *S. dysenteriae* type 1

### Cathelicidin-mediated killing

The killing of *Shigella* spp. with CAP-18 has been described earlier (18). Bacterial suspension (2×10^2^ cfus) was used with different dilutions of LL-37 (human cathelicidin) (Innovagen, Lund, Sweden) or CAP-18 (rabbit cathelicidin) (Innovagen). Control wells contained MHB alone, bacteria in MHB, and LL-37 (or CAP-18) in MHB. From individual wells, the mixture was plated on MacConkey agar for bacterial colony counts.

### Western blot

Epidemic and endemic *S. dysenteriae* type 1 cells (1×10^9^) were sonicated to lyse the bacteria, and complete killing was verified by plating on agar plate. Sodium dodecyl sulphate-polyacrylamide gel electrophoresis (SDS-PAGE) was performed at 100 V, 200 mA, and at room temperature to separate the lysates. About 2.5 μg of bacterial lysate was mixed with sample buffer (0.0625 M Tris, pH 6·8), boiled for two minutes at 95 °C, loaded on 12% SDS polyacrylamide gel and electrophoresed. Biotinylated molecular weight marker (SDS-PAGE standards, high-range BioRad) was included. Samples were pretreated with proteinase K (10 mg/mL, Sigma-Aldrich for 2-2:30 hours) to digest the proteins/peptides and confirm that the bands obtained were truly protein and not lipopolysaccharide (LPS). Separated proteins/peptides were blotted onto nitrocellulose membrane (Osmonics Inc, Cole-Parmer). Blots were blocked in 1% bovine serum albumin-PBS, washed with 0.1% BSA-PBS, the membrane was incubated sequentially with primary antisera (1:150) (pooled endemic or epidemic rabbit antisera), secondary antibody anti-rabbit IgG (1:200 in 0.1% BSA in PBS-Tween), and streptavidin conjugated with horseradish peroxidase. Membrane was developed with 4-chloro-naphthol to visualize immunoreactivity.

### Statistical analysis

The SigmaStat software (version 3.1) (Systat Software, Inc., Point Richmond, CA) and the SPSS software for Windows (version 12.0) (SPSS, Inc, Chicago, IL, USA) were used for analysis of data. Student's *t*-test or Mann-Whitney rank sum test was used for comparing the immune responses (phagocytosis, oxidative burst, and cathelicidin-mediated killing) between endemic and epidemic strains. Shigellacidal activities of different antisera were compared by Kaplan-Meier survival curves using the log-rank (Mantel-Cox) test. One-way analysis of variance (ANOVA) was used for analyzing the serum shigellacidal responses among *Shigella*-infected patient samples over days. Data are expressed as mean±sandard error of mean (SEM). P value was significant when ≤0.05.

## RESULTS

### Serum shigellacidal response

**Fig. 1.**
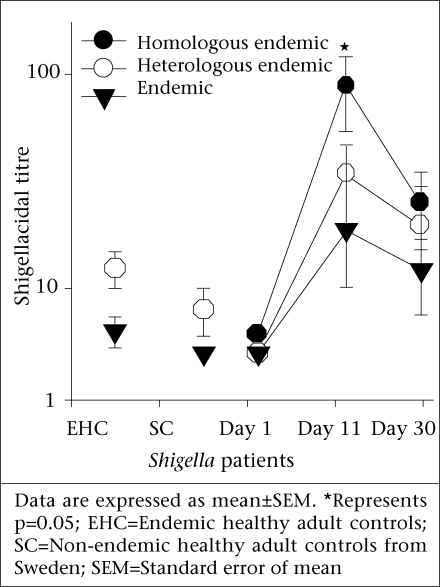
Serum shigellacidal titre of *Shigella* infected patients (day 1, 11, and 30, n=8), endemic healthy adult controls (EHC, n=15), and non-endemic healthy adult controls from Sweden (SC, n=5) against endemic homologous (closed circles), endemic heterologous (open circle), and epidemic strain (closed triangles1 of *Shigella dysenteriae* type 1

Shigellacidal assays were performed to see whether the epidemic and endemic strains had different susceptibility to antibody-mediated killing. The shigellacidal response of patient sera against the respective homologous *S. dysenteriae* type 1 strains was significantly higher on day 11 compared to day 1 (p=0.05) (Fig. 1); the response reduced on day 30 but still remained higher than day 1. The serum shigellacidal response against the heterologous strains had a similar trend but the difference was not significant (Fig. 1). The serum shigellacidal responses of EHC (n=15) and SC (n=5) against the epidemic strain (T2281) and the three endemic strains were comparable.

**Fig. 2.**
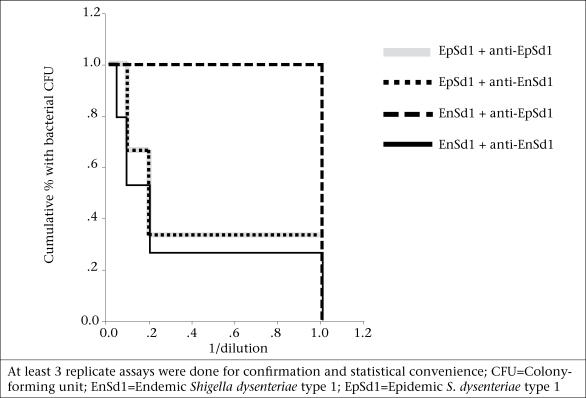
Comparison by Kaplan-Meier analysis of shigellacidal response of rabbit anti-EnSd1 (191316) and anti-Epsd1 (T2218) antisera against 191316 and T2218 strains in different combinations

In the Kaplan-Meier survival plot analysis, rabbit antisera raised with endemic *S. dysenteriae* type  1 showed shigellacidal activity at 1:20 dilution against homologous and at 1:10 dilution against heterologous endemic strains (n=3) and the epidemic strain (Fig. 2 and Table 2). Epidemic antisera showed shigellacidal activity at 1:10 dilution against the homologous epidemic strain but could only kill the endemic strains when used in neat concentration (Table 2). Data are shown in Fig. 2 and Table 2 for one endemic strain only (191316).

### Phagocytic responses and oxidative burst in monocytes and granulocytes

**Fig. 3.**
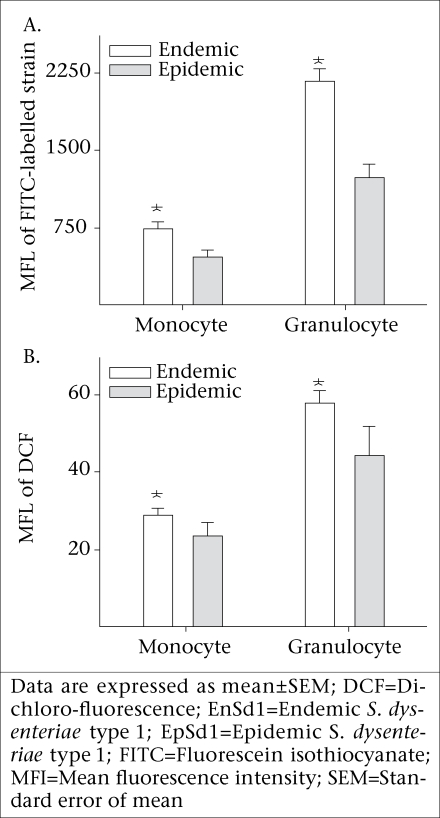
Endemic and epidemic *Shigella dysenteriae* type 1 strain induced (A) phagocytosis responses in monocytes and granulocytes (n=8) against FITC-labelled *Shigella* measured by flow cytometry and (B) oxidative burst responses in monocytes and granulocytes (n=8) measured by the MFI of DCF

To understand whether there were differences in susceptibility of epidemic and endemic *Shigella* strains to innate cellular responses of granulocytes, phagocytosis and oxidative burst responses were studied. Compared to the endemic 191316 strains, the epidemic T2218 strain showed significantly lower susceptibility to phagocytosis by both neutrophils (p<0.01) and monocytes (p<0.01) (Fig. 3A). There was no apparent difference between the epidemic and the endemic strains in inducing oxidative burst response in both monocytes and granulocytes (Fig. 3B).

### *In vitro* killing by cathelicidin

Cathelicidin, a family of classical antimicrobial peptides, kills bacteria by upsetting the integrity of bacterial membrane (19). To check whether cathelicidin exhibited differences in killing of the epidemic and endemic strains due to any outer-membrane structural differences, *in vitro* killing experiment was conducted using human cathelicidin LL-37 and rabbit cathelicidin CAP-18. Both epidemic strain (T2218) and endemic strains (n=3) were completely killed by LL-37 at a concentration of 5.55 μM (minimal inhibitory concentration). Similarly, no differences were found for CAP-18-mediated killing where the minimal inhibitory concentration was 0.9 μM against both epidemic and endemic strains.

### Western blot analysis

**Fig. 4.**
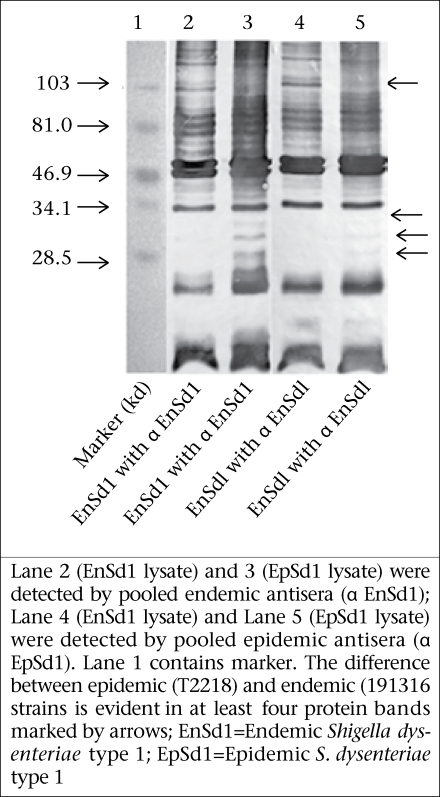
Western blot analysis of sonicated *Shigela* bacterial lysate using rabbit antisera raised against endemic (EnSd1, 191316) and epidemic (EpSd1, T2218) strains

To study if the observed differences in immune responses evoked by the epidemic and endemic strains is linked to variations in immune recognition of protein antigens between the strains, Western blot analysis was conducted. Results of analysis revealed that the endemic (191316) and epidemic (T2218) strains differed in protein/peptide complexity and in intensity of some protein/peptide bands. Both endemic and epidemic antisera recognized three antigens (30-35 kd) in the epidemic strain (Lane 3 and 5 of Fig. 4) but not in the endemic strain. Conversely, both antisera could detect one antigen (~103 kd) only in the endemic strain (Lane 2 and 4, Fig. 4). Proteinase K treatment of the antigen lysate showed no bands in the blot reflecting that these were protein/peptide bands only.

## DISCUSSION

The results of the study showed that the epidemic strain of *S. dysenteriae* type 1 mounted lower levels of shigellacidal serum antibodies and was more resistant to phagocytosis by neutrophils and monocytes compared to the endemic strains. Some difference in immunoreactive protein/peptide bands, both in term of complexity and intensity between the epidemic T2218 strain and the endemic 191316 strains, was obvious as detected by Western blot analysis. Interestingly, cathelicidin-mediated killing of the epidemic and endemic strains showed no difference.

A major obstacle in developing an appropriate vaccine against *Shigella* species is the production of protective neutralizing antibodies after immunization that are serotype-specific (20). In line with this, we found that the bactericidal capacity of serum from *S. dysenteriae* type 1-infected patients against homologous *Shigella* increased significantly after two weeks that declined to the control levels after a month. This was similar to antibody-secreting cell (ASC) response after natural shigellosis or vaccination (21) while *Shigella* LPS-specific IgG responses remained elevated in serum for more than six months (22). However, the shigellacidal immune function of patients' antisera was lower against the heterologous *S. dysenteriae* type 1 strains and the epidemic strain (T2218), although all these strains are of the same serotype. Notably, the healthy subjects from *Shigella*-endemic and non-endemic regions did not show significant differences in shigellacidal response against the epidemic and endemic strains. This result further confirms that adaptive immune function in shigellosis is not long lasting and may be one of the reasons why epidemics due to *Shigella* occur successfully in endemic regions in primed population with high baseline IgG antibody titres (22,23).

In rabbits, the shigellacidal antibody response evoked by the epidemic strain was less efficient compared to the endemic strains in killing the strain itself (T2218) and the heterologous endemic strains. This outcome may result from variations in the immunogenicity, suboptimal exposure of peptide antigens of *Shigella* strains and in the affinity and avidity of the specific neutralizing antibodies. One limitation of this study was that the unavailability of patient's serum infected with epidemic strain to compare with endemic patient antisera.

Phagocytes are the effectors of the innate immune system. Human phagocytes, particularly neutrophils, can ingest both opsonized and non-opsonized *Shigella.* Interaction of a phagocyte with a foreign particle ultimately triggers the oxidant cascade that causes structural alterations in the invasive pathogens, irrespective of the type of microbes. There is also a role of opsonization in oxidative burst pathway (24). The rate of phagocytosis of opsonized endemic strain increased by approximately two-fold compared to opsonized epidemic strain. However, we did not find any significant differences in oxidative burst function. The findings indicate greater resistance of epidemic strain to phagocytosis compared to endemic strain but not to oxidative burst-mediated killing. The observed difference in the internalization of the epidemic and endemic strains by blood phagocytes might be explained by the differences in antibody-mediated opsonization of the strains.

Western blot analysis of bacterial lysate using rabbit antisera, raised against the endemic or epidemic strains, revealed some differences in protein/peptide band sizes and also in band intensity between the endemic and the epidemic strains (Fig. 4). The differences in protein/peptide bands clearly indicate the variation in immunogenic properties of the two strains. This variation may give the epidemic strain an edge above the endemic strains in evoking pro-inflammatory responses and severity of the disease during epidemics. For example, the ability of bacterial protiens, such as *S. flexneri* porins to activate Toll-like receptors (TLR-2), thereby stimulating the production and release of proinflammatory cytokines, adhesion molecules, and nitric oxide, suggests a possible role of these bacterial proteins in inflammation (25). Many proteins of the epidemic *S. dysenteriae* type 1, such as type III secretion system, energyy metabolism, acid resistance may also play a significant role in the increased virulence of the epidemic *S. dysenteriae* type 1 strain (26).

Antimicrobial peptides of the cathelicidin family are cationic and amphipathic molecules that bind to the negatively-charged cell membrane of bacteria and kill them by disruption of bacterial membrane integrity possibly through the formation of toroidal peptide-lipid pores (19). LL-37 binds strongly in a dose-dependent fashion to LPS from gram-negative bacteria or to peptidoglycan, lipoteichoic acid, and wall teichoic acid from gram-positive *Staphylococcus aureus* (27). Thus, the lipid components of the bacterial cell envelope seem to play a major role in the antimicrobial activity of LL-37 where surface proteins may not play a significant role. In our study, *in vitro* shigellacidal concentration of cathelicidin (LL-37 or CAP18) was the same for both epidemic and endemic strains, indicating similarities in net charge and hydrophobicity of the outer membrane of both the strains.

### Conclusions

The findings of the study suggest that the epidemic *Shigella* strain is more resistant to antibody-mediated killing and ingestion by phagocytes compared to the endemic strains. The difference in protein/peptide antigens may partially reflect the difference in immune responses evoked by the epidemic and endemic strains. More detailed studies of the varying protein antigens among strains will resolve many unidentified mechanisms influencing the immune responses, specially the levels and types of antibodies. Reduced susceptibility to antibody-dependent killing by the hypervirulent *Shigella* bacteria may be one of the strategies to escape host defense and cause epidemics.

## ACKNOWLEDGEMENTS

The study was supported by the Swedish Agency for Research Cooperation with Developing Countries (Sida/SAREC agreement support; Grant No. 2002-2004) and icddr,b. icddr,b acknowledges with gratitude the commitment of Sida/SAREC to the icddr,b's research efforts. icddr,b also gratefully acknowledges the following donors which provide unrestricted support: Australian Agency for International Development (AusAID), Government of the People's Republic of Bangladesh, Canadian International Development Agency (CIDA), Swedish International Development Cooperation Agency (Sida), and the Department for International Development, UK (DFID). The authors gratefully acknowledge the healthy laboratory personnel who donated blood.
